# OTUD7B stabilizes estrogen receptor α and promotes breast cancer cell proliferation

**DOI:** 10.1038/s41419-021-03785-7

**Published:** 2021-05-25

**Authors:** Jianing Tang, Zeyu Wu, Zelin Tian, Wei Chen, Gaosong Wu

**Affiliations:** Department of Thyroid and Breast Surgery, Zhongnan Hospital of Wuhan University, Wuhan, China

**Keywords:** Breast cancer, Cell growth

## Abstract

Breast cancer is the most common malignancy in women worldwide. Estrogen receptor α (ERα) is expressed in ∼70% of breast cancer cases and promotes estrogen-dependent cancer progression. In the present study, we identified OTU domain-containing 7B (OTUD7B), a deubiquitylase belonging to A20 subgroup of ovarian tumor protein superfamily, as a bona fide deubiquitylase of ERα in breast cancer. OTUD7B expression was found to be positively correlated with ERα in breast cancer and associated with poor prognosis. OTUD7B could interact with, deubiquitylate, and stabilize ERα in a deubiquitylation activity-dependent manner. Depletion of OTUD7B decreased ERα protein level, the expression of ERα target genes, and the activity of estrogen response element in breast cancer cells. In addition, OTUD7B depletion significantly decreased ERα-positive breast cancer cell proliferation and migration. Finally, overexpression of ERα could rescue the suppressive effect induced by OTUD7B depletion, suggesting that the ERα status was essential to the function of OTUD7B in breast carcinogenesis. In conclusion, our study revealed an interesting post-translational mechanism between ERα and OTUD7B in ERα-positive breast cancer. Targeting the OTUD7B–ERα complex may prove to be a potential approach to treat patients with ERα-positive breast cancer.

## Introduction

Breast cancer ranks the most prevalent cancer and the second leading cause of cancer-related death in women worldwide^1^. Based on the expression of estrogen receptor (ER), progesterone receptor (PR), and human epidermal growth factor receptor 2 (HER-2), breast cancer can be divided into at least three subtypes: luminal, HER2-enriched, and triple-negative, which exhibit different histopathological features and treatment sensitivities^2^.

Approximately 70% breast cancer cases are positive for estrogen receptor alpha (ERα), which is known as the biomarker and one of the most successful molecular targets for endocrine therapy^3,4^. It is structurally organized into several domains: transactivation domains AF-1 and AF-2, which recruit both transcriptional coactivators and corepressors; the DNA-binding domain (DBD), which is required for the specific binding to estrogen response elements (ERE) in enhancers or promoters, and the ligand-binding domain (LBD), which is recognized by the 17 beta estradiol hormone (E2)^5,6^. ERα plays a central role in the signal transduction pathways of breast cancer cells, and upregulation of ERα is associated with the initiation and progression of breast cancer^7,8^. The activity of ERα is essential for cell cycle progression in that it accelerates the G1–S-phase transition. Overexpression of ERα promotes breast cancer cell growth by increasing the expression level of oncogenic proteins, including cyclin D1 and c-myc^9^. Since ERα and its signaling pathways are essential in the development of breast cancer cells, detection of ERα is considered as an important indicator for implementation of endocrine therapy^10^. Despite patients with ERα-positive tumors having high sensitivity to endocrine therapy, 30–50% of them will suffer from later relapse and develop acquired resistance. Distant metastases and resistance to therapy are the major causes of death in breast cancer patients^11–13^. Acquired resistance to endocrine therapy is a common problem in ERα-positive breast cancer, while the mechanisms underlying this resistance are not completely defined^14–17^. Thus, it is of utmost importance to understand the dysregulation of ERα signaling.

ERα can be modified by several kinds of post-translational modifications such as ubiquitination, SUMOlyation, and phosphorylation^18–20^. Recent studies have indicated that many proteins and mechanisms are involved in the regulation of ERα stability via the ubiquitin–proteasome system (UPS). Certain E3-ubiquitin ligases, including BRCA1, BARD1, E6AP, CHIP, MDM2, and SKP2, can increase the polyubiquitination to ERα lysine residues and induce its degradation by the 26 S proteasome^21–26^. Several E3 ligases are demonstrated to facilitate ERα signaling via stabilizing ERα protein. RNF31, SHAPRIN, TRIM11, and RNF8 enhance ERα stability by inducing its monoubiquitination^27–30^. TRIM56 stabilizes ERα targeting via promoting K63-linked ubiquitination on ERα protein^31^. In addition to E3-ubiquitin ligases, deubiquitinating enzymes (DUB) also modulate the stability of ERα protein in breast cancer, which functions to cleave ubiquitin chains from ERα proteins to modulate its degradation. A previous study demonstrated that USP7 is a DUB of ERα and promoted breast cancer progression^32^. However, the DUB responsible for ERα deubiquitination is largely unknown.

In the present study, we screened a DUB siRNA library and found that OTUD7B was a possible deubiquitinase responsible for ERα deubiquitination and stabilization in ERα-positive cancer. We also found that OTUD7B promoted cell proliferation and migration through ERα. Overall, our study has demonstrated that OTUD7B is a novel deubiquitinating enzyme of ERα and may prove to be a potential target for breast cancer intervention.

## Results

### OTUD7B depletion inhibits ERα signaling pathway activity

**Fig. 1:**
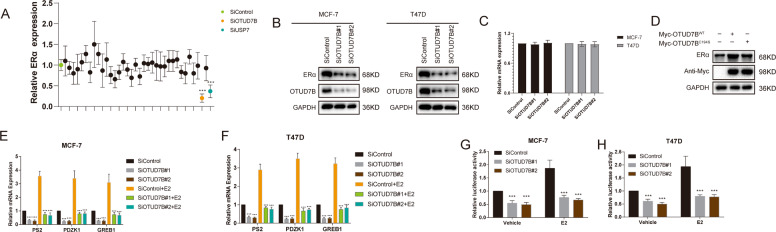
OTUD7B depletion decreases ERα signaling activity in breast cancer cells. **A** The siRNAs specific to each deubiquitinating enzyme were transfected into MCF-7 cells. After 48 h, cells were lysed and the ERα protein level was analyzed by Western blot. Relative ERα protein level was normalized to GAPDH. **B** OTUD7B depletion decreased ERα protein level. **C** OTUD7B depletion did not affect ERα–mRNA level. **D** OTUD7B WT or C194S was transfected into MCF-7 cells and ERα expression was detected. **E**, **F** OTUD7B depletion decreased ERα target genes in the absence or presence of estrogen. Breast cancer cells were transfected with siOTUD7B or siControl. After 48 h, cells were treated with either ethanol or 10 nM estrogen for 6 h. Total RNA was prepared, and the expression of the endogenous ERα target genes, PS2, GREB1, and PDZK1, was determined by qRT-PCR. **G**, **H** OTUD7B depletion affected ERE-luciferase activity. Breast cancer cells were transfected with siOTUD7B or siControl together with ERE-luciferase reporter plasmid. Cells were treated with 10 nM estrogen or vehicle. Luciferase activity was measured 48 h after transfection. *P < 0.05, **P < 0.01, ***P < 0.001.

We initially utilized a siRNA screen library to identify the deubiquitinating enzymes responsible for ERα deubiquitination and stabilization in ERα-positive breast cancer. Four nonoverlapping siRNA mixtures specific for each of the DUBs were transfected into MCF-7 cells; it was found that silencing OTUD7B markedly decreased ERα (Fig. 1A). We then depleted OTUD7B with two nonoverlapping siRNAs separately in MCF-7 and T47D cells to further validate the function of OTUD7B in regulating ERα; as shown in Fig. 1B, OTUD7B depletion significantly decreased the ERα protein levels. Consistently, ectopic expression of OTUD7B profoundly upregulated ERα. While the catalytically inactive mutant C194S (OTUD7B ^C194S^) lost its ability to upregulate ERα, suggesting that OTUD7B regulated ERα in a DUB-activity-dependent manner (Fig. 1C). We examined the expression of ERα target genes (PS2, PDZK1, and GREB1) and found that depletion of OTUD7B dramatically decreased the transcripts of PS2, PDZK1, and GREB1 in both estrogen and vehicle conditions (Fig. 1D, E). In addition, we measured the ERα-luciferase reporter gene activity by OTUD7B depletion to determine whether OTUD7B depletion affected the ERα transcriptional activity It was found that depletion of OTUD7B decreased the ERα-luciferase reporter gene activity in the presence or absence of estrogen (Fig. 1F, G). Overexpression of WT OTUD7B in MCF-7 cells increased the transcripts of ERα target genes and the ERα-luciferase reporter gene activity (Fig. S1). All these results demonstrated that OTUD7B was a regulator of the ERα signaling pathway.

### OTUD7B is associated with ERα protein levels in human breast cancer samples and poor prognosis

**Fig. 2:**
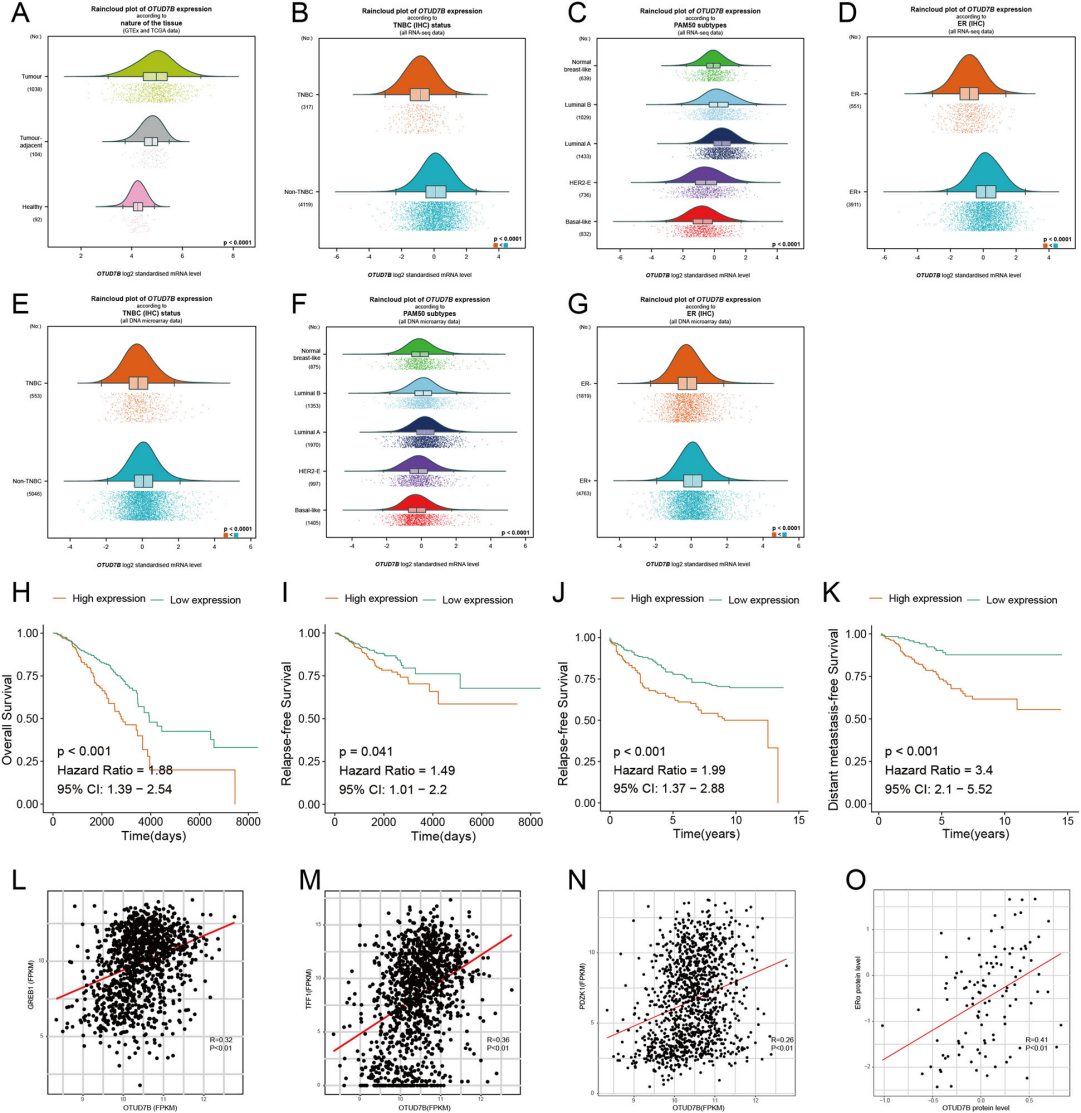
OTUD7B is overexpressed in breast cancer and correlates with poor prognosis. **A**–**G** Expression of OTUD7B in breast cancer. **A**–**D**: Patients were extracted from TCGA BRCA dataset. E–G: Patients were extracted from microarray datasets. All data are available at bc-GenExMiner v4.5(http://bcgenex.centregauducheau.fr/BC-GEM/GEM-Accueil.php?js=1). The significance of differences was calculated using one-way ANOVA test. **H**, **I** OTUD7B is associated with poor overall survival and relapse-free survival of breast cancer patients in TCGA. Cox proportion hazards model was used to understand the significance between two groups. **J**, **K** OTUD7B was associated with poor relapse-free survival and distant metastasies-free survival of ERα-positive breast cancer patients in GSE6532. Cox proportion hazards model was used to clarify the significance between two groups. **L**–**N** Correlations between OTUD7B and ERα target genes in TCGA (Pearson correlation). **O** Correlations between OTUD7B and ERα protein levels in CPTAC (Pearson correlation). *P value < 0.05, **P value < 0.01, ***P value < 0.001.

**Fig. 3:**
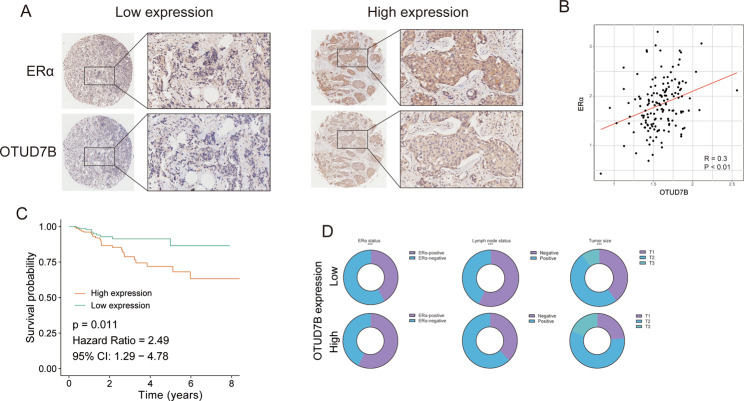
OTUD7B correlates with ERα protein levels and poor prognosis in human breast cancer samples. Tissue microarray (HBreD140Su04) was obtained from Shanghai Biochip Company Co., Ltd, Shanghai, China. The tissue microarray contained 140 breast cancer specimens. **A** The typical staining of ERα and OTUD7B in breast cancer specimens. Specific primary antibodies against ERα (Proteintech, China) and OTUD7B (Proteintech, China) were used for IHC. **B** ERα positively correlated with OTUD7B in breast cancer samples (Pearson correlation). **C** High expression of OTUD7B was associated with poor prognosis. Cox proportion hazards model was used to understand the significance between two groups. **D** OTUD7B expression was associated with the ERα status, lymph-node status, and tumor size. The characteristics were compared between OTUD7B low-/high-groups using chi-square or Fisher’s exact tests. *P value < 0.05, **P value < 0.01, ***P value < 0.001.

OTUD7B expression was analyzed in breast cancers using bc-GenExMiner v4.5 (http://bcgenex.centregauducheau.fr/BC-GEM/GEM-Accueil.php?js=1), a statistical mining tool of published annotated breast cancer transcriptomic data (DNA microarrays [*n* = 10716] and RNA-seq [*n* = 4716]). As shown in Fig. 2, OTUD7B was highly expressed in breast cancer samples, especially in the luminal A subtype (Fig. 2A–G). Then we performed survival analysis of OTUD7B based on TCGA, and found that OTUD7B expression was a poor prognostic factor for breast cancer patients (Fig. 2H, I). As OTUD7B was upregulated in ERα-positive breast cancer patients and associated with the ERα protein level, we then analyzed its prognostic value in ERα-positive breast cancer from GES6532, and observed that high expression of OTUD7B was associated with poor prognosis of patients with ERα-positive breast cancer (Fig. 2J, K). We further analyzed the correlation between OTUD7B and ERα target gene expressions. From TCGA database, we observed that OTUD7B was positively correlated with PS2, PDZK1, and GREB1 expression (Fig. 2L–N). Based on the global proteome data of 105 TCGA breast cancer samples from the Clinical Proteomic Tumor Analysis Consortium (https://cptac-data-portal.georgetown.edu/cptacPublic/), we also observed a positive correlation between OTUD7B and ERα protein levels (Fig. 2O). Consistently, immunohistochemistry (IHC) analysis of two tissue microarrays (TMA) indicated a positive correlation between OTUD7B and ERα staining, and high expression of OTUD7B indicated a poor prognosis (Fig. 3). Further analysis demonstrated that OTUD7B expression was correlated with the ERα status, the lymph-node metastasis status, and tumor size.

### OTUD7B interacts with ERα

**Fig. 4:**
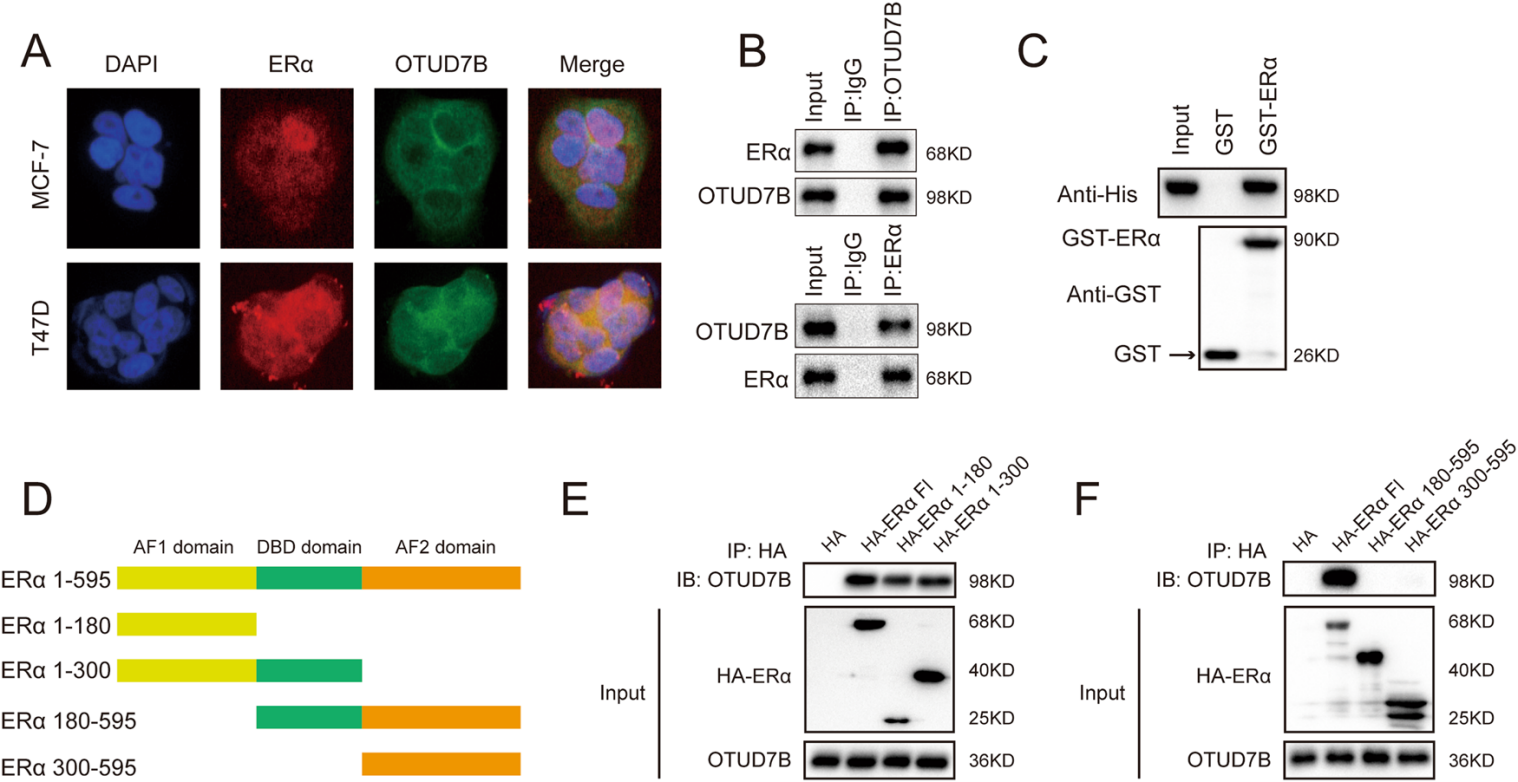
OTUD7B associates with ERα. **A** An immunofluorescence assay demonstrated that OTUD7B and ERα at least partially colocalized in MCF7 and T47D cells. **B** Co-IP assay revealed an association between endogenous OTUD7B and ERα in MCF-7 cells. MCF-7 cells were harvested with RIPA lysis buffer. Co-IP was performed using antibody as indicated. **C** Purified His-OTUD7B was incubated with GST- ERα or GST protein. The interacted OTUD7B was detected via western blot. **D** ER alpha domain structure and deletion mutants used in the study. **E**, **F** OTUD7B interacted with ERα through its AF1 domain. HEK293 cells were transfected with 2 μg Myc-OTUD7B together with HA- ERα full length or mutants. After 24 h, cells were harvested with NP-40 lysis buffer. Co-IP was performed using Myc antibody. The possible interacted ERα domains were detected by HA antibody.

The results of immunostaining demonstrated that ERα and OTUD7B colocalized both in the nucleus and cytosol of breast cancer cells (Fig. 4A). We found that endogenous OTUD7B coimmunoprecipitated with endogenous ERα in the co-immunoprecipitation (Co-IP) experiment (Fig. 4B). GST pull-down assay showed that OTUD7B directly interacted with ERα in vitro (Fig. 4C). Additionally, deletion analysis demonstrated that OTUD7B physically interacted with the AF1 domain of ERα (Fig. 4D, E).

### OTUD7B deubiquitylates ERα

**Fig. 5:**
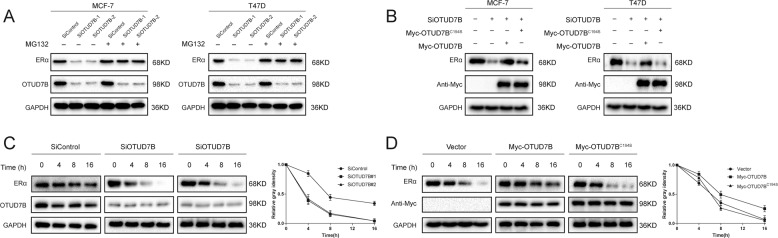
OTUD7B increases ERα stability. **A** In the presence of the proteasome inhibitor MG132, depletion of OTUD7B did not further decrease the ERα protein level. Breast cancer cells were transfected with siOTUD7B or siControl. After 48 h, cells were treated with 10 µM MG132/vehicle for 6 h; cell lysates were prepared for western blot analysis. **B** MCF-7 cells were transfected with OTUD7B (wild type or C194S) together with OTUD7B siRNA. The ERα levels were measured. **C** OTUD7B depletion decreased ERα half-life in breast cancer cells. Breast cancer cells were transfected with siOTUD7B or siControl. After 48 h, cells were treated with 100 µM cycloheximide/vehicle for indicated times. Cell lysates were prepared for western blot analysis. **D** OTUD7B^C194S^ did not increase ERα half-life in HEK293 cells. HEK293 cells were transfected with HA-ERα plasmid and Myc-tag, Myc-OTUD7B, or Myc-OTUD7B^C194S^ plasmids. After 24 h, cells were treated with 100-µM cycloheximide/vehicle for indicated times. Cell lysates were prepared for Western blot analysis.

**Fig. 6:**
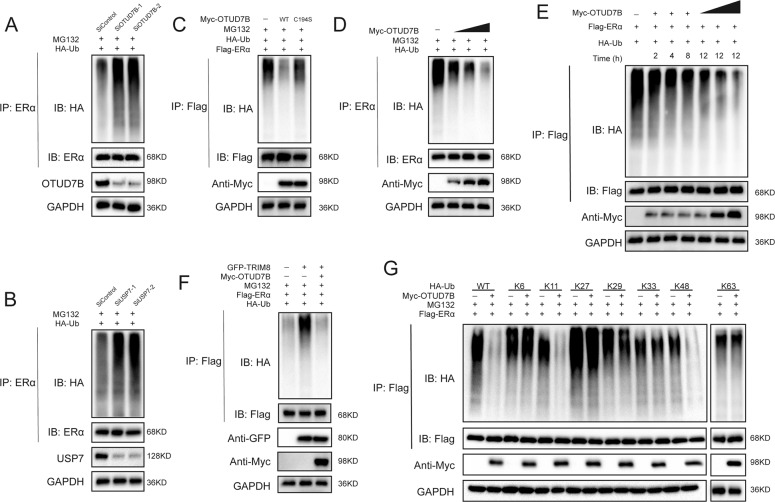
OTUD7B deubiquitylates ERα. **A** MCF-7 cells transfected with the indicated siRNA were treated with MG132 for 6 h before collection. ERα was immunoprecipitated with anti-ERα and immunoblotted with anti-HA. **B** Immunoblotting to detect the ubiquitination of ERα in HEK293 cells cotransfected with Flag-ERα, HA-Ubiquitin, and Myc-OTUD7B (wild type or C194S). **C** Immunoblotting to detect the ubiquitination of ERα in HEK293 cells cotransfected with Flag-ERα, HA-Ubiquitin, and Myc-OTUD7B (wild type or C194S). **D** OTUD7B removed the ubiquitin chain of ERα in a dose-dependent manner. **E** OTUD7B removed the ubiquitin chain of ERα in a time- and dose-dependent manner. **F** ERα ubiquitylation was analyzed in cells transfected with E3 TRIM8 together with OTUD7B or not. **G** HA-WT, K6, K11, K27, K29, K33, and K48 or K63 Ub was cotransfected with Flag-ERα and Myc-OTUD7B into HEK293 cells. After treatment with 10 μM MG132 for 6 h, cell lysates were subjected to ubiquitination assay and the ubiquitination level of ERα was detected by HA antibody. *P value < 0.05, **P value < 0.01, ***P value < 0.001.

The interaction between OTUD7B and ERα suggested that ERα might be a substrate of OTUD7B, and therefore we evaluated the possibility of ERα deubiquitylation by OTUD7B. It was found that OTUD7B deletion dramatically decreased ERα protein level, and this effect could be reversed by addition of the proteasome inhibitor MG132 or overexpression of OTUD7B-WT, but not its catalytically inactive mutant OTUD7B^C194S^ (Fig. 5A, B). We then treated cells with the protein synthesis inhibitor cycloheximide to prove that OTUD7B affected ERα stability. The stability of ERα was significantly decreased in cells depleted of OTUD7B (Fig. 5C). In cells overexpressing OTUD7B-WT, but not OTUD7B^C194S^, the half-life of ERα was prolonged (Fig. 5D). Depletion of OTUD7B significantly increased the level of ubiquitinated ERα in MCF-7 cells. We also observed that USP7 depletion induced ERα ubiquitination (Fig. 6A, B). Conversely, ectopic expression of OTUD7B-WT, but not OTUD7B^C194S^, markedly decreased ERα ubiquitylation in cells both in vivo and in vitro (Fig. 6C and Fig. S2). In vivo deubiquitylation assays showed that OTUD7B directly removed the ubiquitin chain of ERα in a time- and dose-dependent manner (Fig. 6D, E). Furthermore, OTUD7B decreased the ERα ubiquitylation induced by the E3 ligase TRIM8 (Fig. 6F)^33^. We further performed ubiquitination assay with a series of ubiquitin mutants to investigate which type of ubiquitin chain of ERα was deubiquitylated by OTUD7B. It was found that OTUD7B efficiently removed the K11- and K48-linked ubiquitin chain on ERα (Fig. 6F). Taken together, OTUD7B was identified as a specific DUB, which depolyubiquitylated and stabilized ERα.

### OTUD7B regulates cell proliferation and migration through ERα

**Fig. 7:**
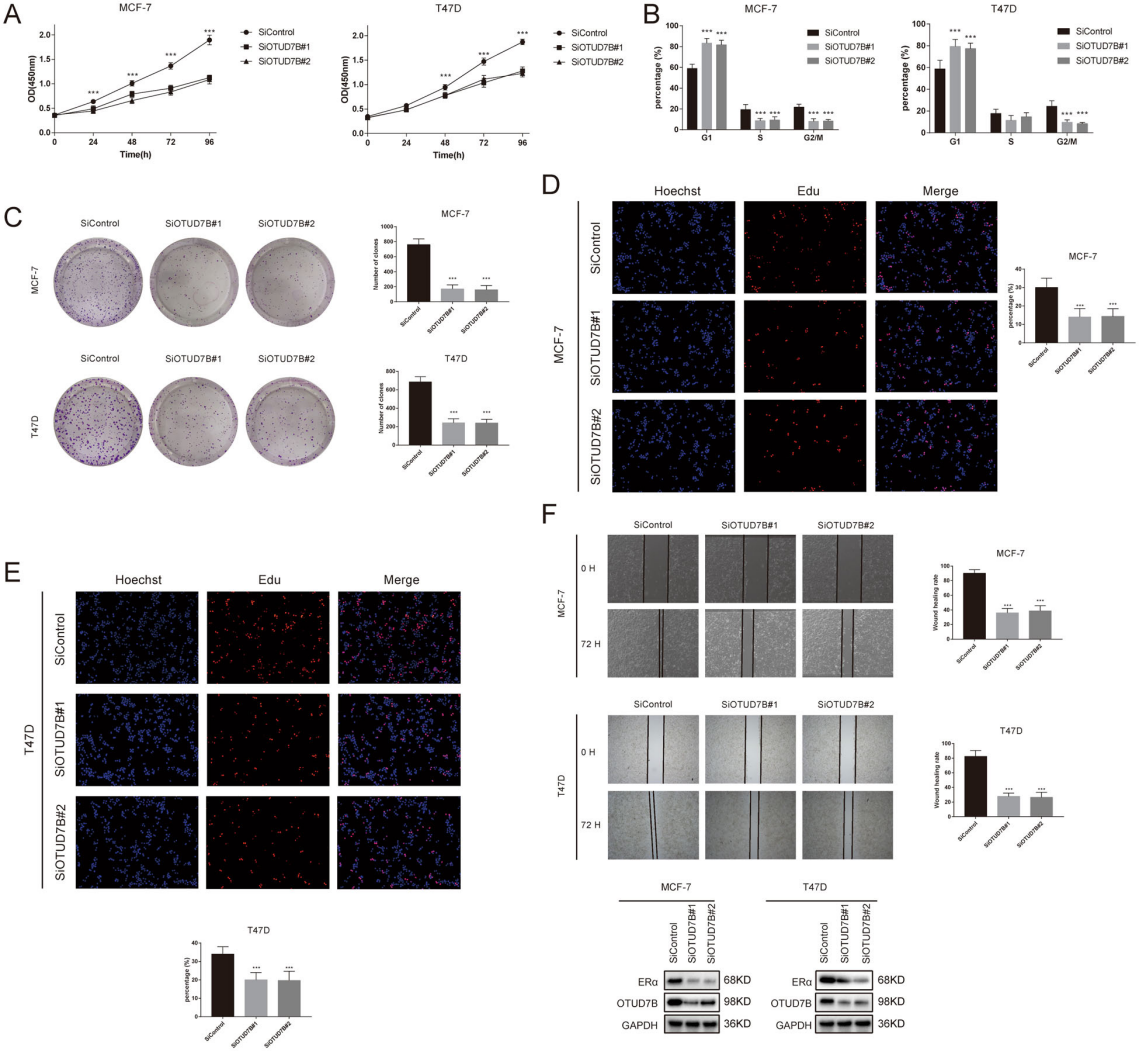
OTUD7B depletion inhibits ERα-positive breast cancer cell proliferation and migration. **A** OTUD7B depletion inhibited cell proliferation in breast cancer cells. **B** OTUD7B depletion induced G1 cell cycle arrest in breast cancer cells. **C** OTUD7B depletion decreased clone formation capability of breast cancer cells. **D**, **E** Representative images of EdU assay of breast cancer cells. **F** Wound-healing assay of breast cancer cells. MCF-7- and T47D cells were transfected with indicated 50 nM OTUD7B siRNA or 50 nM control siRNA. Twenty four hours after transfection, cells were seeded into 6-well plates with 1% FBS with 100% confluence. A straight scratch was made on the cell layer with a yellow pipette tip. Quantification of wound closure was measured every 24 h, and the ERα protein level was measured at the endpoint. *P value < 0.05, **P value < 0.01, ***P value < 0.001.

**Fig. 8:**
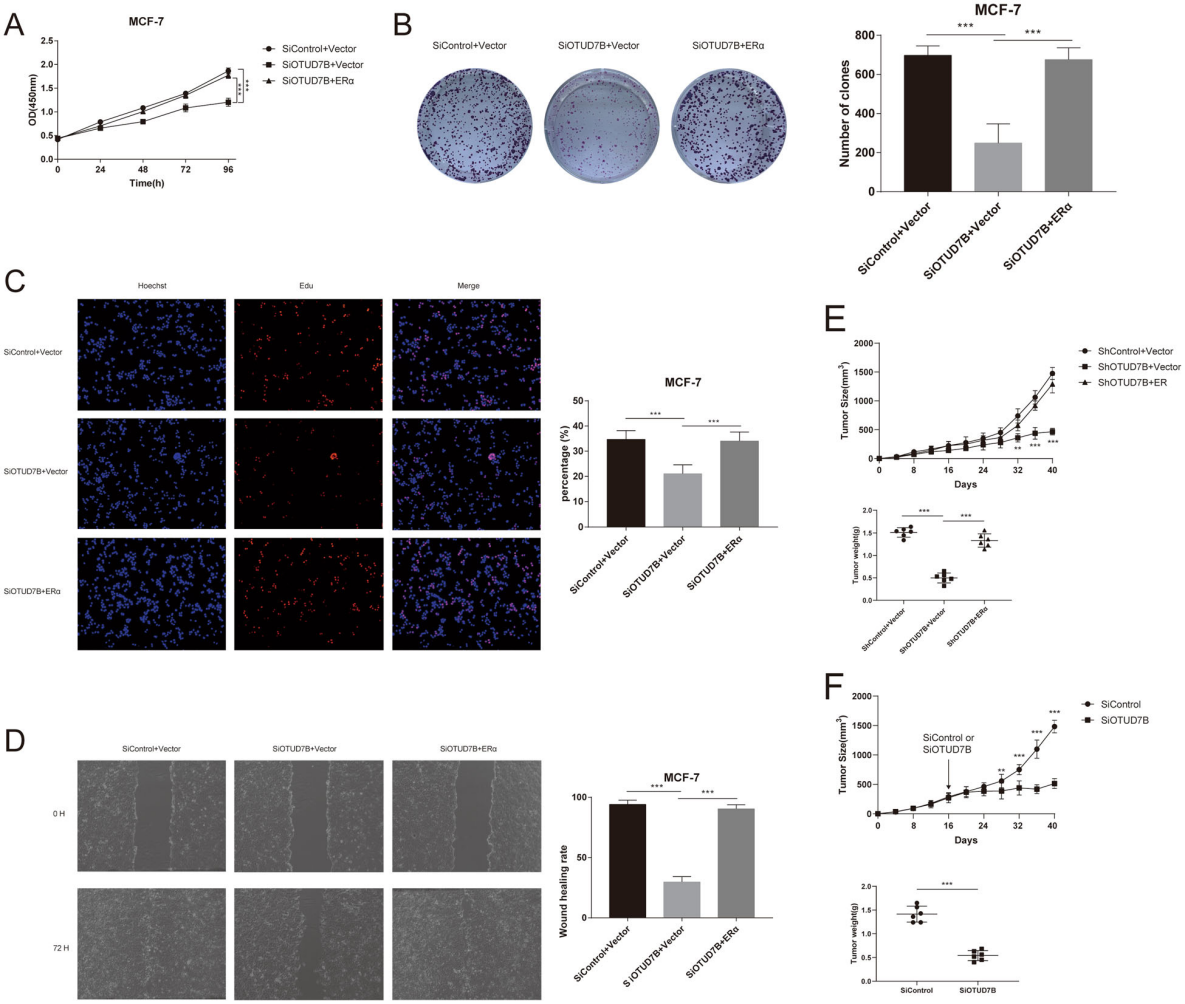
Increased ERα expression reverses the effect of OTUD7B depletion. **A** Cell proliferation assay of MCF-7. **B** Clone formation assay of MCF-7. **C** Representative images of EdU assay of breast cancer cells. **D** Wound-healing assay of MCF-7. **E** Overexpression of ERα in OTUD7B-knockdown cells recovered tumor growth in vivo. **F** In total 2 × 10^6^ MCF-7 cells were injected into the mammary fat pad of female BALB/c nude mice. When the tumor had grown to an appropriate volume (200 mm^3^), the tumor-bearing mice were randomly divided into groups. Cholesterol-modified OTUD7B siRNA or control siRNA (Ribobio, 10 nmol/kg) dissolved in diluted water were intratumorally injected every 3 days until the end of the experiment. *P value < 0.05, **P value < 0.01, ***P value < 0.001.

We next examined the role of OTUD7B in regulating breast cancer progression. Our results demonstrated that depletion of OTUD7B significantly decreased cell proliferation and increased the population in G1 phases, indicating that OTUD7B may regulate G1-to-S transition in ERα-positive breast cancer cells (Fig. 7A, B). The results of clone formation assay revealed that OTUD7B depletion dramatically decreased the clone formation capability in MCF-7 and T47D cells (Fig. 7C). Consistently, EdU incorporation assay indicated that DNA synthesis was inhibited in MCF-7 and T47D cells treated with OTUD7B siRNAs (Fig. 7D, E). Furthermore, depletion of OTUD7B significantly decreased cell migration capacity as revealed by wound-healing assay (Fig. 7F). We then expanded the experiments using stable knockdown cell lines, and USP7 was used as the positive control. As shown in Fig. S3, stable knockdown of OTUD7B or USP7 markedly inhibited breast cancer cell proliferation and migration, reduced tumor growth and metastasis in vivo (Fig. S3). To determine the mechanism of OTUD7B in regulating breast cancer cell proliferation and migration by stabilizing ERα, we performed rescue experiments by ectopic-expressing ERα in OTUD7B- knockdown MCF-7 cells. CCK8 assay indicated that overexpression of ERα largely recovered the proliferation ability of MCF-7 cells (Fig. 8A). Increased ERα expression reversed the clone formation ability of MCF-7 cells (Fig. 8B). Consistently, ERα overexpression also facilitated the DNA synthesis in MCF-7 cells depleted with OTUD7B (Fig. 8C). Wound-healing assay showed that the suppressive function induced by OTUD7B depletion was largely reversed by ERα overexpression (Fig. 8D). Knockdown of OTUD7B significantly inhibited tumor growth in vivo, while the restoration of ERα expression abolished the inhibition induced by OTUD7B depletion (Fig. 8E). In addition, depletion of OTUD7B by using in vivo-optimized RNAi also significantly reduced tumor growth (Fig. 8F). To further confirm that ERα is required for OTUD7B to promote breast cancer cell proliferation and migration, we overexpressed OTUD7B in ERα-depleted cells. We found that OTUD7B could not promote the proliferation and migration of MCF-7 cells depleted with ERα (Fig. S4). These results indicated that OTUD7B promoted breast cancer cell proliferation and migration, at least partially, via the regulation of ERα.

## Discussion

Breast cancer is the most common cancer in women worldwide. Seventy percent of cases are ERα positive^34^. ERα belongs to the nuclear receptor superfamily of transcriptional factors, which was originally cloned from MCF-7 cell in 1985^35^. Targeting ERα has been demonstrated to be one of the most successful strategies for endocrine therapy in ERα-positive breast cancer patients because of its sensitivity and effectiveness^36^. However, endocrine resistance remains an important concern in breast cancer therapy, and several confirmed and hypothetical mechanisms of endocrine resistance have been reported. Single-strand conformation polymorphism (SSCP) reveals a 42-base-pair replacement in exon 6 of the estrogen receptor complementary DNA of a tamoxifen-resistant tumor^37^. In addition to ERα gene mutations in AF-2 domain, breast cancer cells can get endocrine resistance through several kinds of ERα modifications, such as phosphorylation, acetylation, and ubiquitination. P300 could directly acetylate the ERα at lysine residues within the ERα hinge/ligand-binding domain and subsequently promote the ER-signaling activity. Substitution of these residues with charged or polar residues can enhance ERα hormone sensitivity^38^. ERα phosphorylation at certain sites can have a profound impact on ERα function in breast cancer. For example, the phosphorylation at Y537 site changes helix loop conformation and then enhances ligand binding/coactivator-binding efficiency^39,40^. Accumulating evidence has indicated that ER signaling and turnover is tightly linked to the ubiquitin–proteasome system. However, studies exploring the DUB responsible for ERα are limited. In this study, we identified that OTUD7B, which was highly expressed in human breast cancer samples, was a novel ERα co-regulator through post-translational modification. OTUD7B was associated with ERα and inhibits ERα polyubiquitination and degradation. In particular, OTUD7B is essential in breast carcinogenesis.

OTUD7B, a DUB belonging to A20 subgroup of ovarian tumor (OTU) protein superfamily, was previously described as a cell cycle-regulated DUB. OTUD7B can remove K11-linked ubiquitin chains from APC/C substrates at the exit of mitosis by binding, deubiquitinating, and stabilizing the anaphase-promoting complex/cyclosome (APC) substrates, thus contributing to mitotic progression and proliferation^41^. OTUD7B has a preference to remove K11-linked ubiquitin chains from substrates, other studies also suggested that OTUD7B deubiquitinated K48- and K63-linked ubiquitin chains^42–44^. OTUD7B negatively regulated noncanonical NF-κB pathway by deubiquitinating K48-linked polyubiquitin chains of TRAF3, an inhibitor of the NF-kappa-B pathway, thereby preventing TRAF3 proteolysis and overactivation of noncanonical NF-κB^45^. OTUD7B also enhanced EGF-induced growth and migration signals by mediating the deubiquitination of EGFR^46^. Interestingly, OTUD7B regulated the HIF transcriptional activity via directly affecting HIF-1a protein degradation in a proteasome-independent way, possibly through chaperone-mediated autophagy^47^. Besides, OTUD7B had been demonstrated to be able to stabilize Sox2 and maintain neural progenitor cell property through the removal of ubiquitin from Sox2^48^. Although previous evidence has shown that OTUD7B plays a crucial role in carcinogenesis, the molecular mechanisms of OTUD7B participation in the progression of breast cancer remain elusive.

OTUD7B is overexpressed in the breast cancer tissue as compared with that in the paired adjacent normal tissue, and high expression of OTUD7B is associated with poor prognosis of breast cancer patients^46^. In the present study, we first found that OTUD7B was a novel modulator in controlling ERα ubiquitination and stability, which depends on its DUB activity. Depletion of OTUD7B significantly decreased the ERα protein level and inhibited the ERα signaling activity. Based on the analysis of public data available in bc-GenExMiner, we found that OTUD7B was highly expressed in breast cancer tissue, especially in the ERα-positive subtype, and that high expression of OTUD7B was associated with poor prognosis. Consistently, we observed an intimate correlation between OTUD7B expression and ERα protein level according to tissue microarray staining from 140 human breast cancer samples. In addition, survival analysis indicated that OTUD7B expression is associated with poor clinical outcomes. We further explored the molecular mechanism of OTUD7B in regulating ERα, and found that ERα protein level was significantly decreased upon OTUD7B depletion. In addition, ectopic expression of OTUD7B profoundly upregulated ERα in a dose-dependent manner. While the catalytically inactive mutant lost its ability to upregulate ERα. When cells were treated with the CHX, the half-life of ERα was significantly shortened in cells depleted of OTUD7B, but prolonged in cells overexpressing OTUD7B. We then tested whether ubiquitin–proteasome system (UPS) was required to ERα degradation induced by OTUD7B depletion, and found that MG132 largely recovered the decreased ERα expression induced by OTUD7B silence. We also identified that OTUD7B co-located with ERα. The Co-IP experiment demonstrated that endogenous OTUD7B coimmunoprecipitated with endogenous ERα. GST pull-down assay showed that OTUD7B interacted with ERα in vitro in a DUB-activity-dependent manner. The present study also demonstrated that OTUD7B removed the K11- and K48-linked ubiquitin chain from ERα, thus inhibiting proteasome-mediated ERα degradation. In addition, catalytically inactive mutant of OTUD7B (C194S) did not regulate the level of ubiquitination on ERα, suggesting that OTUD7B-promoted ERα stability was a consequence of the enzymatically active site of OTUD7B-catalyzed ERα deubiquitination. Our data further demonstrated that OTUD7B depletion dramatically decreased the proliferation and migration of ERα-positive breast cancer cells. The suppression effects induced by OTUD7B depletion could be reversed by ERα overexpression. These results demonstrated that OTUD7B promoted breast cancer proliferation and migration through increasing ERα stability.

In the present study, we examined the role of OTUD7B in ERα-positive breast cancer cells and identified OTUD7B as the deubiquitinase to mediate ERα deubiquitination. OTUD7B was shown to associate with ERα protein and prolong its stability via removing the K11- and K48-linked ubiquitin chain from ERα. Our data suggest that OTUD7B may drive breast tumorigenesis via ERα expression. As ERα signaling plays a central role in ERα-positive breast cancer cell proliferation, OTUD7B may be a potential target for breast cancer intervention.

## Materials and methods

### Cell culture

The human breast cancer cell lines MCF-7, T47D, and human embryonic kidney HEK293 cells were purchased from American Type Culture Collection (ATCC). T47D cells were cultured with RPMI-1640 (42401, Life Technologies) supplemented with 10% fetal bovine serum (FBS, Gibco, Life Technologies, 10270) and 2 mM l-glutamine (25030, GE Healthcare Hyclone). MCF-7 and HEK293 were cultured with Dulbecco’s Modified Eagle’s Medium (DMEM) that contains 4 mM l-glutamine and 4,5 g/L glucose (41965, Life Technologies) supplemented with 10% FBS. All cells were cultured at 37 °C in an atmosphere of 5% CO_2_.

### Plasmids and RNA inference

Wild-type (WT) OTUD7B and its inactive mutant plasmids were obtained from Hanbio Biotechnology Co., Ltd. (Shanghai, China). The HA-K6, -K11, -K27, -K29, -K33, -K48, -K63, and -Ub plasmids were acquired from Addgene. Small-interfering RNAs targeting OTUD7B (siRNA-1: 5′-CCGAUUGGCCAGUGUAAUU-3′; 5′-CCGAGUGGCUGAUUCCUAU-3′) were synthesized by Genepharma (Shanghai, China). The ERα full- and its deletion constructs were gifted from Dr. Ting Zhuang and were described in our previous study^30^. Lipofectamine 2000 (Invitrogen, Carlsbad, CA, USA) was used for plasmid transfection according to the manufacturer’s instructions.

### RNA extraction and qPCR analysis

Total RNA was extracted from the cancer cells using the RNeasy plus mini kits (Qiagen, Germany) following the manufacturer’s instructions. Reverse transcription was performed using the PrimeScript RT Master Mix (Takara, Japan). qRT-PCR was performed using the SYBR green mix (Toyobo, Japan) with the CFX96TM Real-time PCR Detection System (Bio-Rad, USA) normalized to 36B4. The 2^−ΔΔCt^ method was used to calculate the relative expression. All assays were performed in triplicates.

### Cell proliferation analysis

The cell proliferation rate was detected using Cell Counting Kit-8 (CCK8) assay at indicated time points according to the manufacturer’s instructions. MCF-7 and T47D were transfected with the indicated siRNA, and 24 h later, cells were digested and 2 × 10^3^ cells were seeded in 96-well culture plates. CCK8 solution reagent was added to each well and incubated for 1.5 h at 37 °C. The absorbance was measured at 450 nm using a microplate reader. For clone formation assay, cells were treated with siRNA targeting OTUD7B for 24 h, digested, and seeded into 6-well plates at a density of 1000 cells per well. After 14-day incubation, cells were fixed with 4% paraformaldehyde and visualized by 0.5% crystal violet staining. EdU incorporation assay was performed using Cell-LightTM EdU Apollo 567 In Vitro Kit (Cat number: C10310-1, RiboBio, Guangzhou, China) according to the manufacturer’s protocol, and images were captured using an Olympus microscope.

### Wound-healing analysis

MCF-7 and T47D cells were transfected with siRNA targeting OTUD7B. When cells reached full confluency, we scratched the cell layer with a 200-μl sterile pipette tip and washed with PBS. Cells were maintained in the medium containing 1% FBS and wound distance was measured every 24 h.

### Animal experiments

For xenograft tumor model, female BALB/c nude mice aged 4 weeks (Central Laboratory of Animal Science, Wuhan University, Wuhan, China) were implanted with slow-release 17-beta-estradiol pellets (0.72 mg/90 days, Innovative Research of America) for one week. Animals were randomly divided into different groups (*n* = 8 per group). Stably transfected MCF-7 cells were suspended in PBS (2 × 10^6^ cells/100 μl) and injected into the mammary fat pad. The tumor volume was measured every 4 days until the end of the experiment. For in vivo lung metastasis assays, 2 × 10^6^ MCF-7 cells were injected into female BALB/c nude mice via the tail vein. The lung metastasis was examined at the endpoint by routine histopathological analysis. The experiments were approved by the Ethics Committee at Zhongnan Hospital of Wuhan University.

### Luciferase assay

The ERE luciferase reporter plasmid was transfected into MCF-7 cells together with the Renilla plasmid. After 24 h, luciferase activity of ERE luciferase reporter was measured using the dual-Luciferase Reporter kit (Promega, Germany) following the manufacturer’s protocol.

### Co-immunoprecipitation assay

Cells were lysed with NP-40 lysis buffer containing a cocktail of protease inhibitors. The total cell lysis was precleared with rabbit IgG for 2 h and subsequently immunoprecipitated with the indicated antibody at 4 °C overnight. Protein A/G PLUS-Agarose beads (Santa Cruz) were then added to the lysates and incubated at 4 °C for 2 h. The immunocomplexes were washed with lysis buffer three times and separated by SDS-PAGE. Immunoblotting was performed following standard procedures.

### GST pull-down assays

Bacterial-expressed GST and GST-ERα bound to glutathione–Sepharose 4B beads (from GE) was incubated with recombinant His-OTUD7B at 4 °C for 2 h. Then the beads were washed with GST-binding buffer. The bound proteins were separated by SDS-PAGE, followed by Western blot with indicated antibodies.

### In vivo deubiquitination assay

In vivo deubiquitination assay was performed in HEK293 and breast cancer cells. HEK293 cells were transfected with HA-Ub, Flag-ERα, Myc-OTUD7B, or Myc-OTUD7B^C194S^ plasmid as indicated for 48 h. After 6-h incubation with 10 μM MG132 (MCE), cells were harvested and immunoprecipitated to isolate HA-ubiquitinated ERα. ERα ubiquitination was detected by Western blotting with the indicated antibodies. In breast cancer cells, HA-Ub plasmid was cotransfected with OTUD7B siRNAs into MCF-7 cells. After 24 h, cells were treated with 10 µM proteasome inhibitor MG132 for 6 h, harvested, and incubated with anti-ERα antibody (CST) for 2 h and protein A/G agarose beads (Santa Cruz) overnight at 4 °C. The immunocomplexes were washed with lysis buffer three times and ERα ubiquitination was detected by Western blotting.

### Western blot analysis

Cells were lysed with RIPA extraction reagent (Beyotime, China) supplemented with protease inhibitors (Sigma-Aldrich, USA). Total protein was separated using 10–12.5% sodium dodecyl sulfate polyacrylamide gel electrophoresis and transferred to 0.45-μm PVDF membrane (Millipore, USA). Primary antibodies were ERα (CST, 8644), OTUD7B (Proteintech, 66276-1-Ig), HA (Proteintech, 51064-2-AP), Myc (Proteintech, 60003-2-Ig), and GAPDH (Proteintech, 60004-1-Ig) antibodies. Bands were visualized using an enhanced chemiluminescence (ECL) kit (Boster, China) and detected by ChemiDoc XRS + Imaging System (Bio-Rad).

### Statistical analysis

Student’s *t* test and one-way ANOVA were used to compare two and more groups respectively. Multiple comparison with Bonferroni correction was performed when appropriate. A *P* value <0.05 was considered as statistically significant and all tests were two-tailed. All statistical tests were performed with Prism 7.0 (GraphPad, USA).

## Supplementary information

Supplementary figure legends

Figure S1

Figure S2

Figure S3

Figure S4
