# Supplementary material for: OTUD7B stabilizes estrogen receptor α and promotes breast cancer cell proliferation
Source: Cell Death Dis. 2021 May 25;12(6):534. doi: 10.1038/s41419-021-03785-7 (PMC8149656; doi:10.1038/s41419-021-03785-7)
Supplement: Supplementary file 1 — Supplementary figure legends [file 41419_2021_3785_MOESM1_ESM.docx]

**Supplementary legend**

**Figure S1. Overexpression of wild type OTUD7B increases ERα signaling activity in breast cancer cells.** (A). Overexpression of wild type OTUD7B increased ERα target genes in the absence or presence of estrogen. Breast cancer cells were transfected with wild type OTUD7B or OTUD7B C194S. After 48 h, cells were treated with either ethanol or 10 nM estrogen for 6 h. Total RNA was prepared and the expression of the endogenous ERα target genes, PS2, GREB1, and PDZK1 were determined by qRT-PCR. (B). OTUD7B depletion affected ERE-luciferase activity. Breast cancer cells were transfected with wild type OTUD7B or OTUD7B C194S together with ERE luciferase reporter plasmid. Cells were treated with 10 nM estrogen or vehicle. Luciferase activity was measured 48 h after transfection.

*, *P value < 0.05; **, P value < 0.01; ***, P value < 0.001.*

**Figure S2.** Wild-type OTUD7B, but not OTUD7B^C194S^ possesses DUB activity towards polyubiquitinated ERα *in vitro.* The reaction was carried out at 37℃ for 2 h in 20 µl reaction buffer (20 mM Tris-HCl, pH 7.2, 5 mM MgCl_2_, 50 mM NaCl, 1 mM 2-mercaptoethanol, 10% glycerol) containing the following components: 0.7 µg of E1, 0.9 µg of E2, 12 µg of HA-ubiquitin, 1 µg of TRIM8, 2 µg GST-ERα and GST-OTUD7B. The reaction was terminated by adding 0.4 ml pulldown buffer (20mM Tris-HCl, pH 7.5, 500mM NaCl, 1% Triton X-100, 0.02% BSA, and 5mM β-mercaptoethanol). After addition of 40 µl of glutathione– Sepharose, the samples were rotated at 4 ◦C for 6 h. The beads were washed with 1ml of pulldown buffer three times. The proteins bound to beads were released by boiling in 50 µl of 2× SDS–PAGE sample buffer for 10 min. The samples were then resolved by 8% SDS–PAGE followed by immunoblot analysis using indicated antibodies.

**Figure S3.** **Stable knockdown of OTUD7B or USP7 inhibits ERα-positive breast cancer cell progression.** (A). Stable knockdown of OTUD7B or USP7 inhibited cell proliferation. (B). Stable knockdown of OTUD7B or USP7 induced G1 cell cycle arrest. (C). Stable knockdown of OTUD7B or USP7 decreased clone formation capability of breast cancer cells. (D, E). Representative images of EdU assay of breast cancer cells. (F). Wound-healing assay of breast cancer cells. MCF-7 and T47D cells were stably knocking-down of OTUD7B or USP7, cells were seeded into 6-well plates with 1% FBS with 100% confluence. A straight scratch was made on the cell layer with a yellow pipette tip. Quantification of wound closure was measured every 24 h, and the ERα protein level was measured at the endpoint. (E). Stable knockdown of OTUD7B or USP7 inhibited tumor growth. (F). Stable knockdown of OTUD7B or USP7 reduced the number of lung metastasis nodules.

*, *P value < 0.05; **, P value < 0.01; ***, P value < 0.001.*

**Figure S4. ERα is required for OTUD7B to promote breast cancer progression.** (A). Cell proliferation assay of MCF-7. (B). Clone formation assay of MCF-7. (C). Representative images of EdU assay of breast cancer cells. (D). Wound-healing assay of MCF-7. (E). Xenograft tumor growth of cells transfected with indicated plasmids.

*, *P value < 0.05; **, P value < 0.01; ***, P value < 0.001.*
