# Supplementary figures and images for: OTUD7B stabilizes estrogen receptor α and promotes breast cancer cell proliferation
Source: Cell Death Dis. 2021 May 25;12(6):534. doi: 10.1038/s41419-021-03785-7 (PMC8149656; doi:10.1038/s41419-021-03785-7)

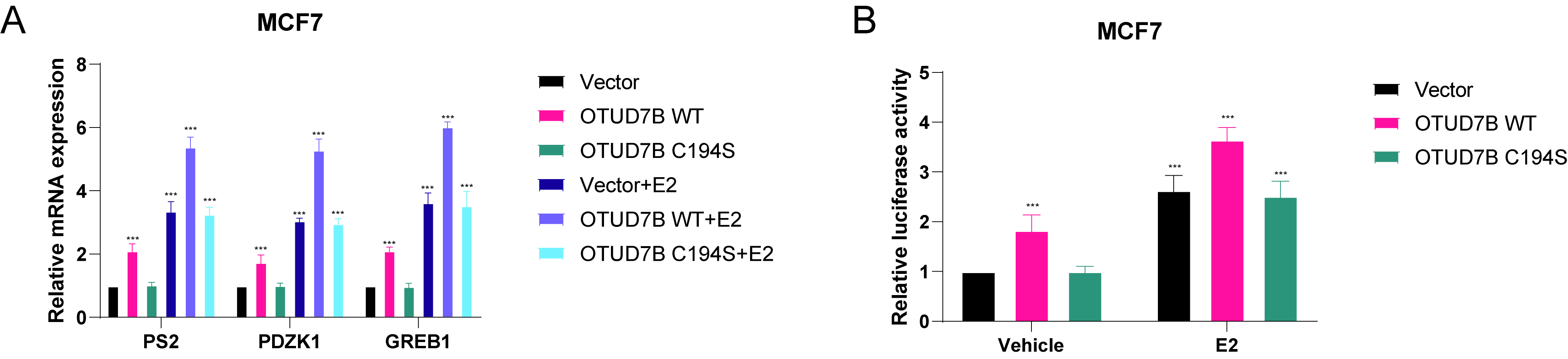

Supplement: Supplementary file 2 — Figure S1 [file 41419_2021_3785_MOESM2_ESM.tif]

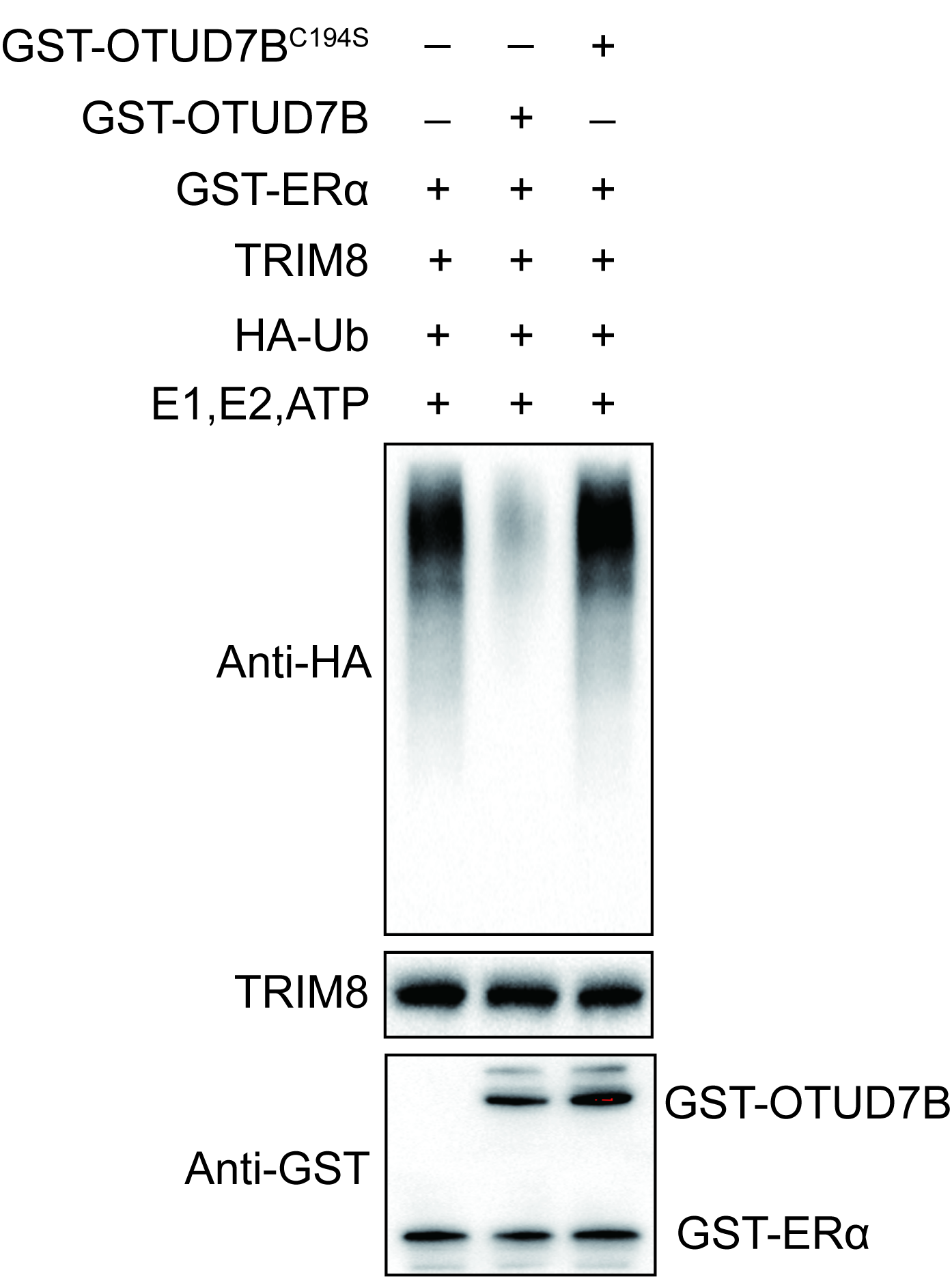

Supplement: Supplementary file 3 — Figure S2 [file 41419_2021_3785_MOESM3_ESM.tif]

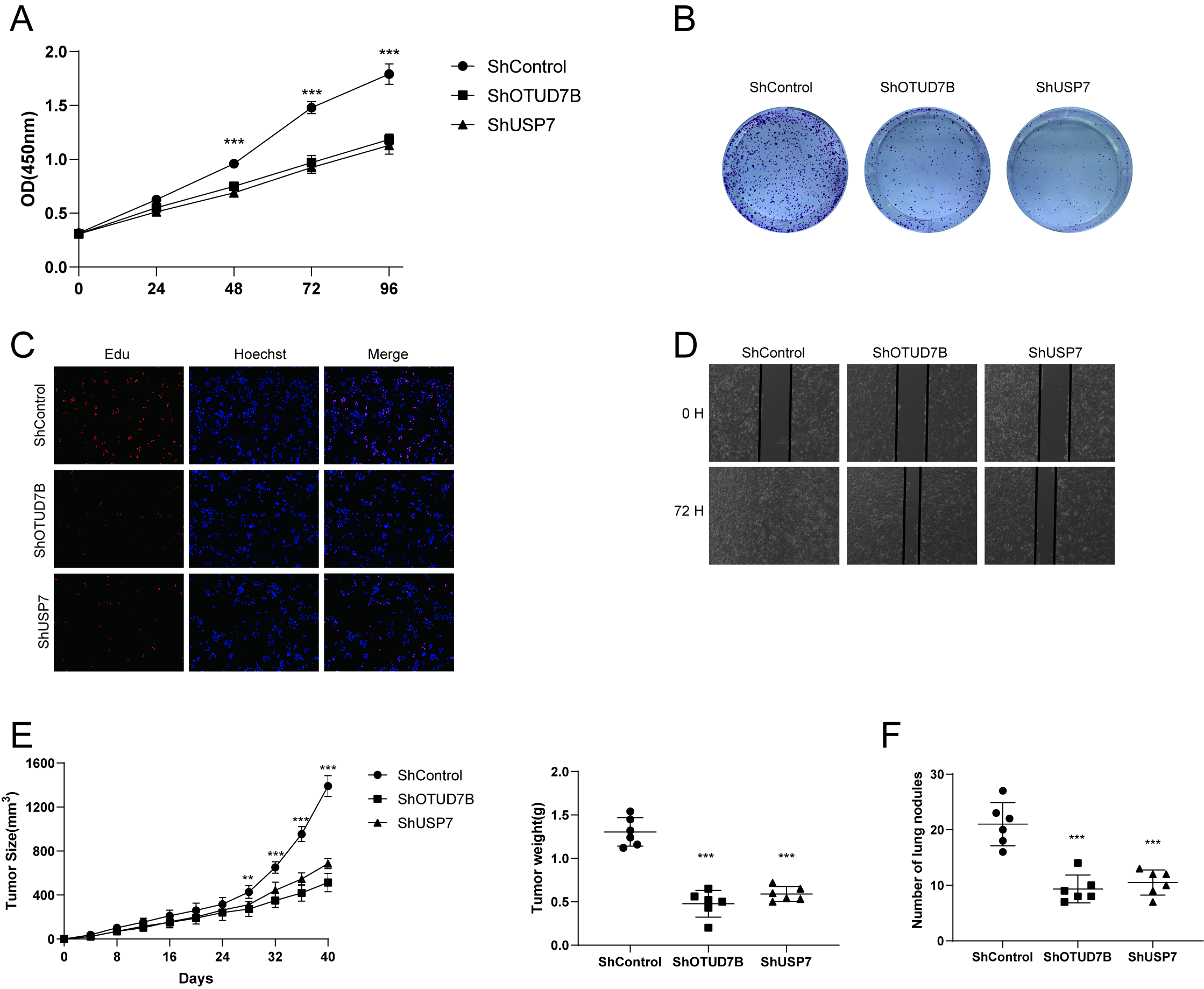

Supplement: Supplementary file 4 — Figure S3 [file 41419_2021_3785_MOESM4_ESM.tif]

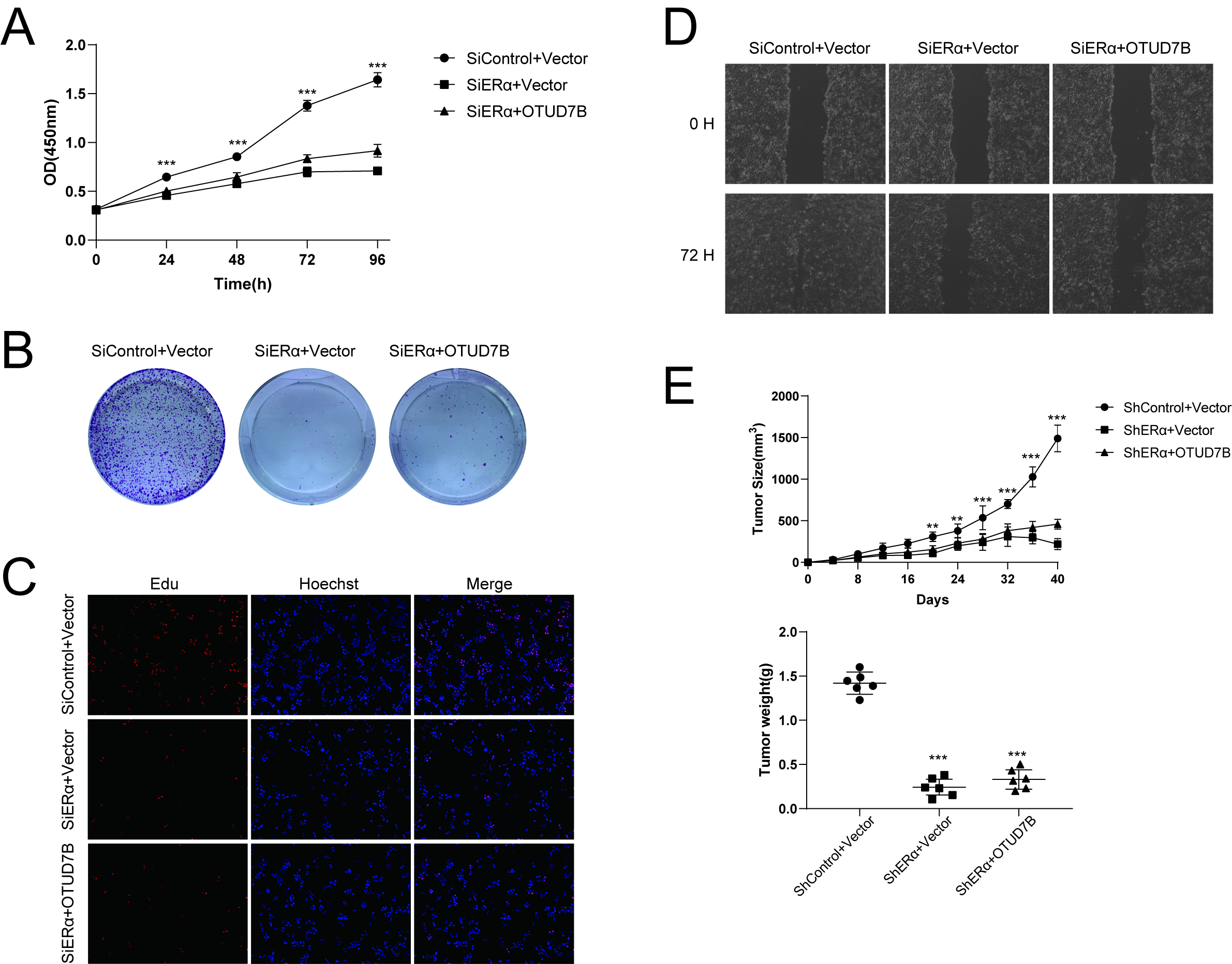

Supplement: Supplementary file 5 — Figure S4 [file 41419_2021_3785_MOESM5_ESM.tif]
